# Cytomegalovirus infection does not impact on survival or time to first treatment in patients with chronic lymphocytic leukemia

**DOI:** 10.1002/ajh.24403

**Published:** 2016-06-01

**Authors:** Helen Marie Parry, Sarah Damery, Christopher Hudson, Matthew J. Maurer, James R. Cerhan, Annette Pachnio, Jusnara Begum, Susan L. Slager, Christopher Fegan, Stephen Man, Christopher Pepper, Tait D. Shanafelt, Guy Pratt, Paul A. H. Moss

**Affiliations:** ^1^ Institute of Immunology and ImmunotherapyUniversity of BirminghamBirminghamB15 2TTUnited Kingdom; ^2^ Institute of Applied Health ResearchUniversity of BirminghamBirminghamB15 2TTUnited Kingdom; ^3^ Faculty of Medicine & Health SciencesUniversity of NottinghamLeicestershireLE12 5RDUnited Kingdom; ^4^ Department of Health Sciences ResearchMayo ClinicRochesterMinnesota; ^5^ Division of Cancer & GeneticsHeath ParkCardiffCF14 4XNUnited Kingdom; ^6^ Department of MedicineMayo ClinicRochesterMinnesota

## Abstract

Human cytomegalovirus (HCMV) is a widely prevalent herpes virus which establishes a state of chronic infection. The establishment of CMV‐specific immunity controls viral reactivation and leads to the accumulation of very large numbers of virus‐specific T cells which come to dominate the immune repertoire. There is concern that this may reduce the immune response to heterologous infections and HCMV infection has been associated with reduced survival in elderly people. Patients with chronic lymphocytic leukemia (B‐CLL) suffer from a state of immune suppression but have a paradoxical increase in the magnitude of the CMV‐specific T cell and humoral immune response. As such, there is now considerable interest in how CMV infection impacts on the clinical outcome of patients with B‐CLL. Utilizing a large prospective cohort of patients with B‐CLL (*n* = 347) we evaluated the relationship between HCMV seropositivity and patient outcome. HCMV seropositive patients had significantly worse overall survival than HCMV negative patients in univariate analysis (HR = 2.28, 95% CI: 1.34–3.88; *P* = 0.002). However, CMV seropositive patients were 4 years older than seronegative donors and this survival difference was lost in multivariate modeling adjusted for age and other validated prognostic markers (*P* = 0.34). No significant difference was found in multivariate modeling between HCMV positive and negative patients in relation to the time to first treatment (HR = 1.12, 95% CI: 0.68–1.84; *P* = 0.65). These findings in a second independent cohort of 236 B‐CLL patients were validated. In conclusion no evidence that HCMV impacts on the clinical outcome of patients with B‐CLL was found. Am. J. Hematol. 91:776–781, 2016. © 2016 Wiley Periodicals, Inc.

## Introduction

Human Cytomegalovirus (HCMV) is a prevalent beta‐herpes virus that is usually asymptomatic upon primary infection. HCMV maintains lifelong latency within cells of the myeloid lineage and its prevalence increases with age 1, 2. The age‐adjusted prevalence in the United States is 50.4% by the age of 50 years 3 and continues to increase with each decade of life 4.

In health, a significant proportion of both CD4+ and CD8+ T cells are required to maintain viral latency and prevent HCMV viral reactivation 5, 6. This extreme expansion of HCMV‐specific T cells, which is most marked in the CD8+ T cell repertoire, contributes to a reduction in the number of naïve T cells and leads to an inversion of the CD4:CD8 T cell ratio 7. There is increasing concern that the burden of CMV infection can lead to health problems, particularly in older people, and CMV seropositivity has been associated with impaired responses to vaccination 8, increasing levels of inflammatory cytokines 9 and an increase in overall morbidity and mortality in several studies 10, 11.

Chronic lymphocytic leukemia (B‐CLL) is characterized by the proliferation of mature B lymphocytes and has a median age at diagnosis of 71 years (https://www.hmrn.org/statistics/incidence; cited 1/6/15). The development of B‐CLL leads to a state of immune suppression and patients exhibit an increased susceptibility to infection from an early stage of disease 12. The mechanisms that underlie the reduction in immune competence are multifactorial but include hypogammaglobulinemia and T cell dysfunction 13. In addition, the burden of chronic infection and changes in the T cell repertoire are also important for overall prognosis in B‐CLL, and an inverted CD4:CD8 T cell ratio is associated with disease progression 14. The CD4:CD8 ratio is markedly lower in CMV seropositive individuals compared with those who remain uninfected, and this effect is particularly profound in patients with CLL. Indeed, one of the paradoxes of CMV infection in patients with CLL is that the magnitude of the virus‐specific immune response is actually higher than in healthy individuals, despite the state of immune suppression, in a mechanism that is believed to be a response to increased levels of subclinical viral reactivation 15, 16. As such there is now considerable interest in how chronic CMV infection may influence the clinical profile of patients with B‐CLL 17. Indeed, if the infection was shown to accelerate the progression of disease then there may be a potential role for the use of anti‐viral medication in disease management 18.

We utilized two large independent prospective cohorts of newly diagnosed patients (the “discovery” and “confirmation” cohorts) to evaluate the relationship between HCMV seropositivity and disease outcome in patients with B‐CLL. In particular we examined the relationship between CMV serostatus and time to treatment (TTT) and overall survival (OS) and show that infection status does not impact significantly on these variables.

## Methods

Patients in the discovery cohort (*n* = 347) were participants in the ongoing prospective cohort study of NHL patients from the Molecular Epidemiology Resource of the University of Iowa/Mayo Clinic Lymphoma Specialized Program of Research Excellence (SPORE). This study was reviewed and approved by the Human Subjects Institutional Review Board at the Mayo Clinic and the University of Iowa, and written informed consent was obtained from all participants in accordance with the Declaration of Helsinki. Since September 2002, enrollment was offered to consecutive newly diagnosed patients with B‐CLL, evaluated at Mayo Clinic Rochester or the University of Iowa within 9 months of diagnosis 19. All patients were US residents aged 18 years and older. Exclusion criteria included known HIV infection and unwillingness or inability to provide written informed consent. Patients fulfilled IWCLL criteria and/or fulfilled the World Health Organization criteria for the small lymphocytic lymphoma variant (SLL) variant of B‐CLL. Baseline clinical, laboratory, and treatment data were abstracted from medical records and participants provided peripheral blood serum samples. All participants were then followed every 6 months for the first 3 years, and then annually thereafter; disease progression and deaths were verified through medical record review.

For the confirmation cohort, newly diagnosed patients with B‐CLL were enrolled from clinics at the University Hospital of Wales and Llandough Hospital. Patients were diagnosed by an experienced hematologist and met the IWCLL criteria for B‐CLL. Informed consent was taken in accordance with ethical approval obtained from the South East Wales Research ethics committee. Samples were taken within 12 months of diagnosis and prior to any treatment. Data for disease progression and mortality was collected on an annual basis and verified through medical records.

### Prognostic parameters

Prognostic testing, including immunoglobulin heavy chain variable [IGHV] region gene mutation analysis, ZAP‐70 status, CD38 status, CD49d status, and cytogenetic abnormalities assessed by interphase FISH testing, were all performed as part of clinical or research studies using methods previously described 20, 21, 22. Adverse FISH results were defined by the presence of 17p deletion or 11q deletion. Serum immunoglobulin measurement was only available at the time of sample collection in the discovery cohort.

### CMV ELISA

HCMV seropositivity and HCMV IgG titer were determined using the previously described CMV enzyme‐linked immunosorbent assay (ELISA) 23. Briefly, mock and viral‐infected lysate was coated onto ELISA plates and incubated overnight. Patient plasma samples and a series dilution of standards (derived from three known HCMV‐positive plasma donors) were added to the plate for 1 hr. The plate was washed three times. An anti‐human IgG‐horseradish peroxidase secondary antibody was then added to the plate for 1 hr. After further washing, TMB (3,3′,5,5′‐tetramethylbenzidine) substrate was added and the plate kept in the dark for 10 minutes before the addition of 1 M HCl. The sample was assessed using an ELISA reader at 450 nm. To determine HCMV titers, mock values were first subtracted from lysate values and titers then calculated against the standard curve. Values greater than 10 were considered to be seropositive. To ensure accuracy, all samples were tested in duplicate.

### Statistical analysis

Chi‐square testing (and Fisher's exact test where appropriate) was used to assess the association between HCMV seropositivity and demographic, clinical and prognostic factors in B‐CLL patients, and both the discovery and confirmation cohorts were characterized using descriptive statistics. For compatibility between datasets, Rai staging for the discovery cohort was converted to Binet staging. Rai 0/1 representing Binet A, Rai stage II representing Binet B, and Rai stage III/IV representing Binet stage C. TTT was defined as the time from diagnosis to disease progression requiring treatment. OS was defined as the time from diagnosis to death due to any cause. Patients without an event or death were censored at time of last known follow‐up. Kaplan–Meier survival curves and Cox proportional hazards regression models were used to assess the association between HCMV positivity and the outcomes of interest. Cox models were adjusted for demographic and B‐CLL prognostic factors which were found to impact significantly on survival and TTT in univariate analysis. An analysis of the continuously distributed CMV IgG ELISA values was also performed. HCMV titer was transformed to the base 2 log and entered as a covariate into cox regression analysis. Analyses were performed using SPSS Version 21 (Armonk NY: IBM Corp), and the threshold for statistical significance was set at *P* = 0.05. CMV ELISA results were analyzed using GraphPad Prism Version 5.03 (GraphPad Software, San Diego, CA), and CMV titers were calculated with reference to the standard curve.

## Results

### Patient cohorts

The discovery cohort consisted of 390 patients, of which 347 patients were identified as having serum available for CMV testing and were used to explore the relationship between CMV and clinical outcome in B‐CLL. Serum samples were collected a median of 2.9 months following diagnosis (range 0–73).

The confirmatory cohort consisted of 236 patients. All patients had serum drawn within 12 months of diagnosis, median 5.8 months (range 0.5–11.75). The clinical characteristics for these two prospective observational cohorts are shown in Table 1.

**Table 1:** Patient Characteristics of the Discovery and Confirmatory Cohorts

	Discovery cohort (*n* = 347)	Confirmatory cohort (*n* = 236)
Median age at diagnosis (years)	62 (37.0–91)	65.1 (24–99)
Follow up time (years + IQR)	2.98 (2.41–4.17)	7 (4–10.2)
Male sex	237 (68%)	150 (64%)
Binet stage A	180 (52%)	182 (77%)
Binet stage B	155 (45%)	25 (11%)
Binet stage C	11 (3%)	27 (11%)
Missing	1	2
CD38 positive	82 (24%)	99 (42%)
CD38 negative	218 (63%)	117 (50%)
CD38 missing	47	20
Zap70 positive	93 (27%)	82 (35%)
Zap 70 negative	203 (59%)	134 (57%)
Zap 70 missing	51	20
IGHV mutated	176 (51%)	125 (53%)
IGHV unmutated	104 (30%)	40 (12%)
IGHV missing	67	71
CD49d positive	64 (18%)	102 (43.2%)
Cd49d negative	131 (38%)	69 (29.2%)
Cd49d missing	152	65
FISH		
Normal	76(22%)	61(26%)
13q−	120(35%)	37(16%)
12+	50(14%)	6(3%)
11q−	22(6%)	15(6%)
17p−	11(3%)	5(2%)
Other	5(1%)	2(0.8%)
Missing	63	110

### CMV status and clinical outcome

#### Discovery cohort

The median follow up time for this cohort was 3 years (IQR 2.4–4.2) and at the point of last data collection 68 participants [20% (68/347)] had died. Of the 347 participants, 198 (57%) were HCMV seropositive and 149 (43%) were HCMV seronegative. HCMV positive patients were significantly older at the time of diagnosis with a median age of 64 years (range 37–87) compared with 60 years in uninfected individuals (range 37–91); *P* < 0.0001. There was no association between HCMV seropositivity and any other demographic or clinical characteristic (Binet stage, sex, ECOG performance score or absolute lymphocyte count) or prognostic markers (ZAP‐70, CD38, IGHV, CD49d, FISH) (Table 2). About 49 deaths (25%) were recorded for the HCMV positive group and 19 deaths (13%) were noted in the HCMV negative group. Of the 68 deaths, 33 were unrelated to CLL and 29 were documented as a CLL‐specific cause of death (7 due to secondary malignancies, 3 due to infection, and 19 from refractory disease). The cause of death was not available for 6 patients.

**Table 2:** Association of CMV with Selected Characteristics of the Discovery Cohort

	CMV negative (*n* = 149) (43%)	CMV positive (*n* = 198) (57%)	*P* value
**Age** Median (range)	60 (37.0–91.0)	64 (37.0–87.0)	*P* < 0.0001
**Gender**			
Female	43 (28.9)	67 (66.2)	*P* = 0.32
Male	106 (71.1)	131 (33.8)	
**Binet stage**			
A	71 (47.7)	109 (55.3)	*P* = 0.32
B	72 (48.4)	83 (42.1)	
C	6 (4.0)	5 (2.5)	
Missing	0	1	
**CD38**			
Positive	34 (25.8)	48 (28.6)	*P* = 0.59
Negative	98 (74.2)	120 (71.4)	
Missing	17	30	
**ZAP70**			
Positive	42 (32.8)	51 (30.4)	*P* = 0.65
Negative	86 (67.2)	117 (69.6)	
Missing	21	30	
**IGHV**			
Mutated	81 (35.2)	95 (61.3)	*P* = 0.55
Unmutated	44 (64.8)	60 (38.7)	
Missing	24	43	
**CD49d**			
Positive	23 (27.7)	41 (36.6)	*P* = 0.19
Negative	60 (72.3)	71 (63.4)	
Missing	66	86	
**FISH**			
Normal	30 (24.4)	46 (28.6)	*P* = 0.21
13q	50 (40.7)	70 (43.5)	
Trisomy 12	25 (20.3)	25 (15.5)	
11q	13 (10.6)	9 (5.6)	
17p	2 (1.6)	9 (5.6)	
Other	3 (2.4)	2 (1.2)	
Missing	26	37	
**Serum IgG**			
N	95	95	*P* = 0.63
Mean (SD)	818.3 (281.3)	854.4 (316.0)	
Median	820	806	
Range	190.0–1750.0	189.0–2220.0	
Missing	54	61	

**Figure 1:**
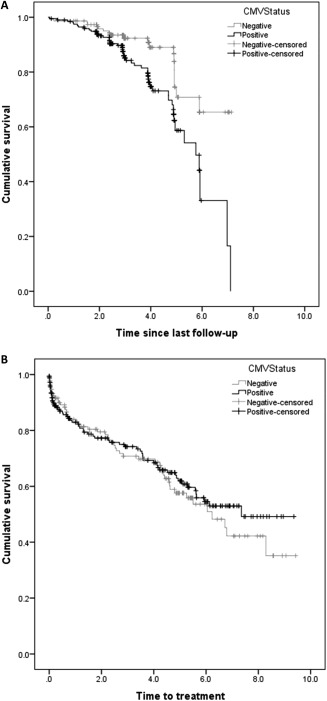
(A) Kaplan–Meier curve for overall survival by CMV status in the discovery cohort is shown. Univariate analysis showed CMV positive patients had significantly inferior overall survival, with a hazards ratio of 2.28 (*P* = 0.0024) However, this significance was lost on multivariate analysis (HR = 0.61, 95% CI: 0.22–1.69; *P* = 0.34) (**B**) demonstrates the time to first treatment by CMV status in the discovery cohort. No significant difference in time to first treatment was observed on multivariate analysis (HR = 1.12, 95% CI: 0.68–1.84; *P* = 0.651).

In univariate analysis HCMV positive patients had significantly inferior overall survival, with the risk of death at any time point more than twice that observed in HCMV negative patients (HR 2.28, 95% CI: 1.34–3.88; *P* = 0.0024) (Fig. 1a). CLL‐specific cause of death was also investigated in relation to CMV serostatus, HCMV positive patients again had a significantly higher risk of dying from CLL‐related causes at any point than HCMV negative patients (HR 3.41, 95% CI: 1.37–8.47, *P* = 0.008).

However, after adjusting for age, Stage, ZAP‐70, FISH, IGHV, CD38, and CD49d in a multivariate survival model, this risk was attenuated, with only age (HR 1.12; 95% CI: 1.06–1.19; *P* < 0.0001) and unmutated IGHV (HR 2.78; 95% CI: 1.07–7.23; *P* = 0.036) remaining significant as independent risk factors for OS. No difference in survival was seen between HCMV positive and negative participants in the multivariate model (HR = 0.61, 95% CI: 0.22–1.69; *P* = 0.34).

We next went on to investigate the relationship between CMV infection and the time to treatment, and this information was available on 322 of the original 349 patients in the cohort. The median time to first treatment was 7.4 years and at the last follow up 115 of the 322 patients (36%) were confirmed to have received treatment for B‐CLL. A log rank test for differences between HCMV positive and negative participants demonstrated no difference in time to first treatment in either a univariate model (HR = 0.90, 95% CI: 0.62–1.30; *P* = 0.560) or a multivariate model adjusted for age, Rai Stage, and prognostic variables (HR = 1.12, 95% CI: 0.68–1.84; *P* = 0.651). Binet stage C compared with stage A (HR = 0.33, 95% CI: 0.01–0.09; *P* < 0.0001) and stage B (HR = 0.11, 95% CI: 0.04–0.28; *P* < 0.0001), as well as expression of CD49d (HR 2.05, 95% CI: 1.16–3.62; *P* = 0.013) remained significant predictors of time to treatment after adjusting for other variables in the multivariate model (Fig. 1b).

#### Confirmation cohort

In order to validate the results found in the discovery cohort, a further large cohort with a longer follow up time was assessed for the same outcome data. The median follow up time of the confirmation cohort was 7 years (IQR: 4–10.2 years). Of 236 patients in this cohort, 109 (46%) had required treatment at the point of last follow up and 93 had died (39%). 179 (76%) of the patients were found to be HCMV seropositive and CMV serostatus was not found to be associated with age at diagnosis within this cohort. No association between HCMV seropositivity and any of the other demographic, clinical, or prognostic markers was found in the confirmation cohort. Of the 93 deaths, 10 were unrelated to CLL and 83 were documented as a CLL‐specific cause of death (3 due to secondary malignancies, 40 due to infection, and 40 from refractory disease).

As noted in the discovery cohort, a shorter median overall survival of 10.6 years was noted in HCMV positive participants (95% CI: 8.4–12.5) compared with 15.9 years (95% CI: 6.9–25.0) in HCMV negative participants although this did not reach statistical significance on either univariate (HR 1.45, 95% CI: 0.86–2.43; *P* = 0.158) or multivariate analysis (HR 0.96, 95% CI: 0.57–1.63; *P* = 0.882). Analysing CLL‐specific cause of death also failed to reveal any significant difference between HCMV positive and negative patients 1.60 (0.91–2.81, *P* = 0.100).

When investigating the influence of CMV infection on TTT in this confirmation cohort, Binet stage, CD38, CD49d, ZAP‐70, IGVH, and adverse FISH status were all found to impact significantly on TTT in univariate testing. Data was complete for prognostic factors in only 68 patients, of which 44 participants required treatment. No association between HCMV status and TTT was found once these prognostic variables were adjusted for in multivariate modeling (HR 1.13, 95% CI: 0.50–2.55; *P* = 0.766). Only Binet stage C compared with stage A (HR 0.43, 95% CI: 0.12–0.14; *P* < 0.0001) and stage B (HR: 0.29 (0.09–0.93); *P* = 0.037) were significant independent predictors of time to treatment.

### CMV titer and clinical outcome

High titers of CMV‐specific antibody have been associated with reduced overall survival in healthy elderly individuals and may represent a response to increased levels of subclinical viral load 24. We investigated this in our cohorts by calculating hazard ratios for the association between patients with B‐CLL and Log2 HCMV titer as a continuous variable for both overall survival and time to treatment. Data were available on 198 HCMV seropositive B‐CLL patients within the discovery cohort. About 49 deaths had occurred within this cohort and although a trend was observed between increasing antibody titer and reduced survival, this did not reach statistical significance (HR 0.95, 95% CI: 0.82–1.09; *P* = 0.460). Similarly, in the confirmation cohort, 75 deaths occurred amongst 179 HCMV positive participants, but no relationship was seen between HCMV titer and OS (HR 1.02, 95% CI: 0.88–1.19; *P* = 0.793). Therefore, no significant relationship was found between level of CMV‐specific antibody and overall survival in CMV positive patients.

Similarly, no significant associations were seen in either cohort between HCMV titer and TTT (Table 3). Given the similar outcome data for HCMV seropositivity on OS and TTT in both cohorts, and in an attempt to increase the number of HCMV positive individuals available for statistical analysis, the datasets were pooled and used to assess whether the titer of HCMV IgG as a continuous variable impacts on overall survival or TTT. Data from 337 HCMV positive individuals were analyzed, of which 124 deaths were recorded during follow up, but again no relationship was found between HCMV titer and overall survival (HR 1.01, 95% CI: 0.91–1.13; *P* = 0.800) or time to treatment (HR 1.00, 95% CI: 0.91–1.10; *P* = 0.972).

**Table 3:** Impact of CMV IgG Titer on Overall Survival and Time to First Treatment

OVERALL SURVIVAL	Median IgG titer (inter‐quartile range)^a^ all patients	Median IgG titer (inter‐quartile range) alive patients	Median IgG titer (inter‐quartile range) deceased patients	Number of deaths in CMV positive individuals (%)	Hazards ratio (95% CI)	*P* value
Discovery cohort	227.1 (113.8–503.4)	234.7 (114.6–556.1)	213.0 (105.2–440.7)	49/198 (25%)	0.95 (0.82–1.09)	0.46
Confirmatory cohort	202.1 (103.9–357.1)	196.9 (110.5–342.2)	209.5 (93.2–404.9)	75/179 (42%)	1.02 (0.88–1.19)	0.79
Combined cohorts	211.9 (106.8–434.6)	211.9 (114.6–448.8)	210.1 (98.3–433.1)	124/377 (33%)	1.01 (0.91–1.13)	0.80
TIME TO TREATMENT	Median IgG titer (inter‐quartile range)^a^ all patients	Median IgG titer (inter‐quartile range) treated patients	Median IgG titer (inter‐quartile range) untreated patients	Number of patients treated in CMV positive individuals (%)	Hazards ratio (95% CI)	*P* value
Discovery cohort	227.8 (118.5–506.5)	234.7 (123.2–455.0)	227.1 (107.0–522.7)	63/185 (34%)	0.99 (0.87–1.13)	0.93
Confirmatory cohort	202.1 (103.9–357.1)	204.0 (95.5–357.4)	185.7 (105.7–363.0)	86/179 (48%)	1.02 (0.89–1.17)	0.78
Combined cohorts	211.9 (107.1–445.6)	213.0 (115.0–418.6)	211.9 (107.1–445.6)	149/364 (41%)	1.00 (0.91–1.10)	0.97

a Titer is the base 2 log, to take account of the non‐linearity of the untransformed variable.

## Discussion

CMV is recognized as an important pathogen in patients who are immune suppressed and viral reactivation is a common occurrence in patients with B‐CLL who undergo treatment with alemtuzumab. However, there is now interest in the potential health associations of chronic viral carriage in a range of clinical settings, particularly in patients with B‐CLL in whom the CMV‐specific T cell immune response can expand to a remarkably high frequency 15, 16. Despite this, no published epidemiological data exists investigating the relationship between CMV and B‐CLL prognosis. This work, reports the prevalence of HCMV within the two cohorts was approximately 60%–70%, which is comparable with studies of viral seroprevalence in healthy people at a similar median age of 64 years 23. As such we find no evidence that CMV infection is associated with the development of B‐CLL. In this regard it is noteworthy that chronic viral infection has been suggested as a potential antigenic stimulus to account for the finding of shared immunoglobulin genes sequences in tumors from different patients. Indeed, the recurrent IGHV1‐69 sequence that is common in B‐CLL has been shown to react with the pUL32 phosphoprotein from CMV 25.

In the discovery cohort HCMV seropositivity was associated with a twofold increased risk of death on univariate analysis. However, this association was lost in multivariable analysis after adjusting for other prognostics variables, the most notable of which was age, as HCMV positive patients were on average 4 years older than the CMV seronegative patients. All other variables tested for within this cohort were not statistically different between CMV positive and negative patients. The prevalence of CMV is known to rise with increasing age and as such it may not be surprising that CMV seropositive patients were somewhat older 24, 26. However, samples were tested at the point of diagnosis, which then raised an alternative possibility that CMV infection could potentially serve to delay the diagnosis of B‐CLL. To address this question it would be important to obtain information on CMV serostatus on a healthy cohort from the same geographical area taken during the same time period but no appropriate cohort was available for comparison. Moreover, no difference was found between age and CMV serostatus in the confirmation cohort, which suggests that a potential protective effect of CMV infection on the diagnosis of B‐CLL is unlikely. This discrepancy between the two cohorts in relation to age and CMV serostatus is likely to reflect the different geographical locations of the two cohorts, as the timing of primary exposure to CMV is known to vary dramatically depending on geographical and socio‐economic factors 27.

The lack of an association between survival and CMV infection in B‐CLL patients contrasts with studies which have investigated the impact of CMV status in healthy elderly individuals. This may be due partly to the younger age of the patients with B‐CLL compared with studies of healthy elderly people, which have focused predominantly on those between 70 and 100 years of age 11, 28, 29. In addition, the detrimental impact of chronic HCMV on the health of older people has been linked with elevated levels of inflammatory markers, including IL‐6 23, 30, 31, which are often already increased in patients with B‐CLL and may therefore confound any impact of HCMV on further health outcomes 32, 33. It may also suggest that any negative effect of HCMV infection in the elderly is outweighed by a diagnosis of B‐CLL. Another possibility that should not be discounted is that CMV infection may actually play a potential beneficial role on immune function in patients with B‐CLL. The virus stimulates a strong Th1 immune response and leads to accumulation of large numbers of cytotoxic cells, including NK and Υδ T cells. CMV infection has been associated with a reduction in disease relapse after stem cell transplantation and may therefore potentially play a role in the setting of other hemopoietic malignancies 34, 35.

In healthy elderly populations a low CD4:CD8 ratio and a high CMV‐specific T cell response both predict for poor survival 36 and it has previously been suggested that CMV seropositivity may impact on poorer survival in B‐CLL, albeit using a small sample size (*n* = 57) and based on univariate analysis only 16. Our current study suggests that HCMV does not impact on survival negatively and although HCMV status was determined around the time of diagnosis in this work, it is unlikely many (if any) patients who were CMV negative at diagnosis would later seroconvert 37. As age can act as a confounding factor for HCMV seropositivity in survival analysis, a larger patient cohort with longer follow up time may be needed to identify if these factors act independently.

In addition, high HCMV‐specific antibody titers have been correlated with poor clinical outcome in elderly donors 28 but we were unable to demonstrate this in patients with B‐CLL. The development of hypogammaglobulinemia is a feature of progressive CLL but unlike other herpes viruses such as VZV and EBV, HCMV‐specific IgG titers have been shown to increase with disease progression and progressive hypogammaglobulinemia, Indeed, in a large cohort of patients with CLL, almost 50% demonstrated an increase in their CMV‐specific IgG titer 4.6 years later and all CMV positive patients were still found to have detectable CMV IgG 37. As such it is unlikely that any patients with hypogammaglobulinemia were incorrectly identified as seronegative for latent CMV. The increase in CMV IgG observed over time may suggest episodes of reactivation are occurring. However, patients with B‐CLL rarely suffer from overt clinical episodes of HCMV reactivation, except after treatment with alemtuzumab or following allogeneic transplantation, suggesting that CMV‐specific T cell function is adequate to prevent clinical viral replication. Indeed, the function of CD8+ HCMV specific T cells is not obviously impaired in patients with CLL 38, 39.

In conclusion, our data provide no evidence that CMV infection can predispose toward the development of B‐CLL. In addition, established infection and HCMV‐specific antibody titer do not appear to have an impact on time to first treatment or overall survival in newly diagnosed patients with B‐CLL.
